# A Scalable Data Access Layer to Manage Structured Heterogeneous Biomedical Data

**DOI:** 10.1371/journal.pone.0168004

**Published:** 2016-12-09

**Authors:** Giovanni Delussu, Luca Lianas, Francesca Frexia, Gianluigi Zanetti

**Affiliations:** Data-Intensive Computing Group, CRS4, Pula, Italy; West Virginia University, UNITED STATES

## Abstract

This work presents a scalable data access layer, called PyEHR, designed to support the implementation of data management systems for secondary use of structured heterogeneous biomedical and clinical data. PyEHR adopts the openEHR’s formalisms to guarantee the decoupling of data descriptions from implementation details and exploits structure indexing to accelerate searches. Data persistence is guaranteed by a driver layer with a common driver interface. Interfaces for two NoSQL Database Management Systems are already implemented: MongoDB and Elasticsearch. We evaluated the scalability of PyEHR experimentally through two types of tests, called “Constant Load” and “Constant Number of Records”, with queries of increasing complexity on synthetic datasets of ten million records each, containing very complex openEHR archetype structures, distributed on up to ten computing nodes.

## Introduction

**Fig 1:**
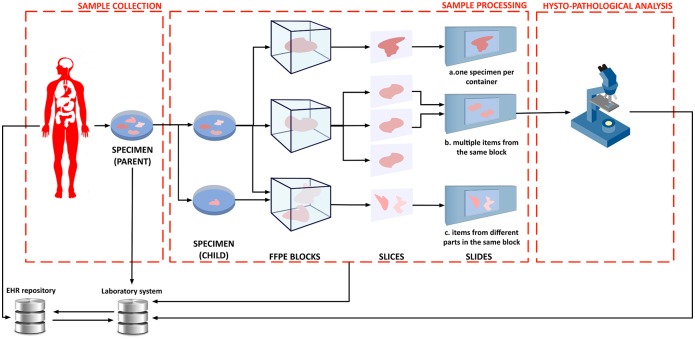
Example of slides acquired for digital pathology. From left to right: a tissue sample extracted from a patient is divided into multiple specimens; these are fixed into containers, blocks of formalin or paraffin; in turn the blocks are sliced. The produced slides can have one specimen per container, multiple items from the same block or items from different parts in the same block. For Figure credits see the Section Acknowledgments.

Next-generation sequencing(NGS) and other high-throughput technologies are rapidly transforming life sciences [1, 2]. Their use is now routine in biology labs and is very quickly expanding to clinical research [3] and applications [4]. Thus, these technologies have abruptly transformed some areas of biology into data-intensive domains, where sophisticated computational analysis is essential to extract biologically relevant information from the raw data [5, 6]. Consequently, a great amount of effort has been expended to develop scalable computational tools that are able to cope with the current data load and with the future, much larger, loads that are expected to arise due to the increasing adoptione of these technologies in large-scale clinical studies [7, 8]. A particular characteristic of these studies is that their outcome depends on complex and deep computational pipelines that are strongly influenced by the choice of parameters and configuration; therefore it is extremely important to fully trace the processing steps performed and to keep these traces as an integral part of the results. This is particularly true now for the analysis of NGS data and it will become even more relevant with the expected diffusion of Computer-Aided Diagnosis systems [9]. Analogously, the explosive diffusion of digital data acquisition for biomedical applications, arising, among other things, from fully traced clinical procedures [10], Internet of Things (IoT) personal health acquisition devices [11, 12] and cyber-physical systems adoption [13–17], has dramatically increased the amount of context information that can be attached to phenotypic information, in addition to the overall system complexity [18–20]. In more general terms, all computed and phenotypic information typically maps to deeply structured records with potentially recurring substructures. An example is shown in Fig 1, which illustrates a conventional slide acquisition pipeline used in the context of digital pathology. The metadata recorded by the pipeline features structure repetition and nesting. The development of novel data analysis algorithms and metadata handling techniques, therefore needs to be complemented by robust, scalable, flexible, computable, uniform and implementation-independent descriptions of deeply structured data [3]. Here, with scalability and flexibility we mean the capability respectively to cope with large amount of data and with its structure and type evolution. The latter issue is typical of longitudinal biomedical studies, which often need to adapt to changing heterogeneous data types because of their long duration compared to the useful life of particular acquisition technologies.

Ideally, common solutions based on ad hoc database tables should be replaced by computable formalisms for the meta-description of structured records. Such systems should easily support operations such as aggregations on sophisticated profile descriptions across all the available records, as well as in-depth, drill-down navigation of all data related to a specific study participant.

In this article, we describe PyEHR, a data access layer for data-management systems for biomedical research, specifically designed to efficiently manage queries on large collections of very heterogeneous structured records with recurring repeated substructures. The system is built for the large data volumes and the evolving and heterogeneous data structures encountered in biomedical research.

Our motivation for the development of PyEHR comes from our direct experience in providing computational support to a wide range of biomedical research projects, including large-scale genome sequencing studies [21–23], safety assessments of novel gene therapy approaches [24], as well as the running of a large scale NGS facility [25].

We use openEHR [26]—a computable formalism for the meta-description of structured clinical records—as a systematic approach to handle data heterogeneity while keeping a clear separation between the well defined semantic description of data structures and their concrete storage implementation. On the other hand, scalability is achieved through a multi-tier architecture with interfaces for multiple data storage systems. With this approach, it is possible to express queries at the semantic level and to perform structural optimizations well before they are translated to a specific implementation. Currently, the specific Database Management Systems (DBMS) supported are two NoSQL databases/search engines: MongoDB [27] and ElasticSearch [28]; support for other DBMS’s, even relational database management systems (RDBMS), can be easily added.

We are interested in traversing data in a heterogeneous but structured data lake [29], efficiently and in a scalable manner; to achieve this efficiency we exploit the structure of the queried data. In practice, our approach is based on indexing each unique class of structures to accelerate the search process and in addition decoupling as much as possible the logical description of the operation that should be performed from the specific data engine that performs it. The specific context in which we consider the problem is, of course, biomedical research but the approach is quite general.

PyEHR has been tested on a set of synthetic data, created to challenge it with very deep and complex structures. As a reference, we performed the same query tests using a straightforward search approach implemented as an Apache Hadoop MapReduce [30] application. Our results show that PyEHR has good scalability characteristics.

### Context

Biomedical research and its clinical applications are quickly becoming data intensive enterprises [31]. On the one hand, this transition is fueled by an increasing data generation capability in this field. It is increasing in part because of technological innovation, resulting in cheaper and faster omics data-generating devices and high-resolution digital imaging devices, as well as the introduction of patient-centric data-generating devices—such as body sensors, smartphone apps, social networks, etc. Furthermore, the data-generating capability is increased by the ongoing adoption of electronic health records (EHR), which result in a more thorough data digitalization and collection. On the other hand, there have been technological advances in storage and computing capabilities too, which make it possible and more accessible to collect, store and analyze increasingly large amounts of data.

Often the biomedically relevant information is obtained as the end result of long multi-step computational pipelines that are in continuous evolution and whose outcomes depend strongly on chosen parameters, configurations, software versions, etc. [32] It is important to note that the metadata defining the pipeline are as important as the results themselves; they are critical to interpreting the different outcomes and to reproducing them.

**Fig 2:**
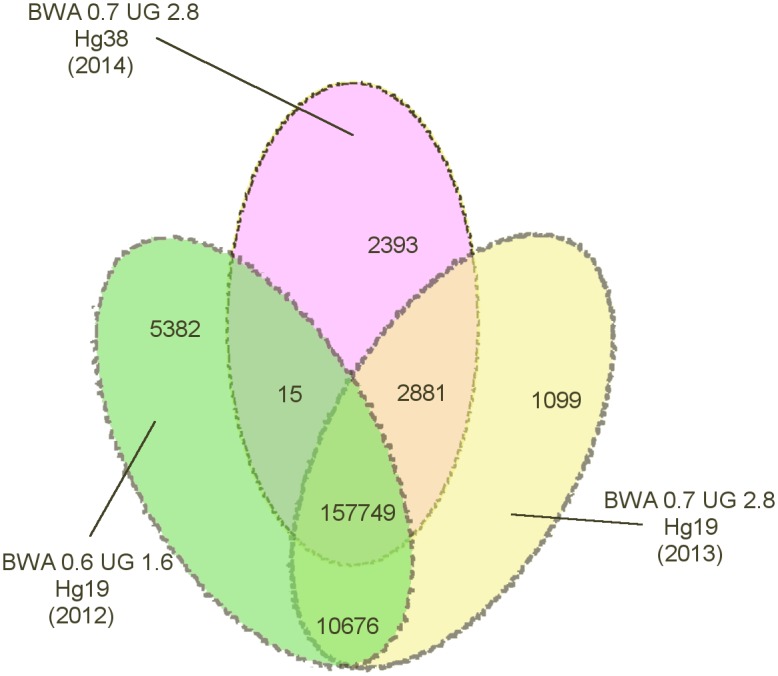
Number of common variants produced by different pipelines given identical input. In the Figure, the underlined text indicates the versions of: the Burrows-Wheeler Aligner(BWA), the Broad Institute’s Genome Analysis Toolkit (GATK) Unified Genotyper(UG) and human genome reference(Hg). For Figure credits see the Section Acknowledgments.

Their importance is exemplified in Fig 2, which highlights the degree of dissimilarity between sets of genomic variants identified from the exome sequencing of parent-child trios [33]. All sets of variants were extracted from the same data using conceptually identical analysis protocols, but using different versions of the same software tools and/or reference genomes. The pattern shown is typical of data-intensive analysis pipelines: the evolution of analysis algorithms and reference datasets has a drastic impact on results. Therefore to understand how old results were obtained or to effectively update them—recomputing them from the original source data using the new version of tools and models—it is critical to have the full provenance information.

**Fig 3:**
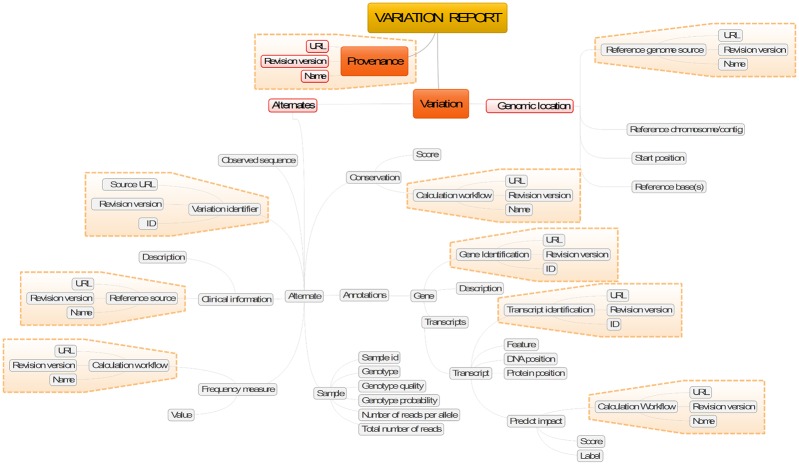
Mind map of a set of annotations produced by a variant calling procedure. Dashed lines highlight recurrent blocks.

Like the process that computes them, genomic variants are also a form of complex data. They comprise deeply structured datasets, as illustrated in Fig 3. It is important to note how the data structure uses recurrent substructures—for instance to annotate variants; in the Figure these are shown with dashed lines.

Moreover, analogously to biomedical data, clinical information is also becoming more structured. Fig 1 shows an example from digital pathology, where the path from data to actionable information is composed by a complex chain of steps that needs to be fully an accurately documented to precisely relate the data—in this case, a whole-slide digital image—to the relevant context. In the example, a tissue sample extracted from a patient is divided into multiple child specimens, which are fixed into paraffin blocks and then sliced. The resulting slides can thus contain one or more slices, which have to be tracked.

In practice, gathering phenotypic information—as raw data and analysis results—leads to the accumulation of vast quantities of heterogeneous data, which can be structured (e.g., reports, analysis provenance graphs), semi–structured (e.g., health care claims, billing records) or unstructured data (e.g., raw data, medical images) [34–36]. Given the continuous evolution of the data sources, it is not technically feasible to map the problem to a data warehouse. On the other hand, data lakes are well suited for this situation [32, 37]. The major advantages of data lakes over the standard data warehouses are their flexibility, as they can contain any type of data—e.g., structured, semi–structured or unstructured– and their robustness, since they only require a schema-on-read, they are easily reconfigured, and they are relatively easy and economical to scale [38].

### Related Work

Even while limiting this discussion to non-commercial products, there are several software packages to manage clinical data [39]. What is lacking, however, is a package aimed specifically at managing large amounts of complex structured clinical and biomedical data. In particular, there are no instruments to store such data while preserving their semantics and provenance information, nor to traverse them “horizontally” to extract population-wide for secondary use. Instead, most of the software available now performs vertical retrieval of medical records per single patient. These software are generally based on relational database management systems which, by their scalability and flexibility characteristics, are not well suited for large collections of complex heterogeneous structured data.

Among the numerous openEHR-based EHR management projects, the ones that are most relevant to this work, in our opinion, are: EHRflex, LiU EEE,ResearchEHR and Ethercis. We briefly discuss them in the following paragraphs.

EHRflex [40, 41] is an archetype-based clinical registry system designed to employ clinical archetypes as guidelines for the automatic generation of web interfaces. It is geared towards clinical use and data collection. It is based on eXist, an open source native XML database, which it uses to store the data it collects. Though this tool has its positive aspects, Miranda et al. tested a number of XML databases [42], including eXist, and found that without further optimizations they are not suitable to be adopted as persistence mechanism for openEHR-based systems used in production if population-wide ad hoc querying is needed—since they are orders of magnitude slower than the relational database to which they were compared, ie, MySQL.

Similarly, LiU EEE [43] is another EHR environment that relies on the XML database eXist and thus suffers from the same limitations as EHRflex. However, LiU EEE is expressly designed for educational and research purposes and not for large-scale analysis. Its main goal is to help newcomers and developers experiment with and learn about the openEHR model, from Archetype Definition Language (ADL) archetype handling [44] to Archetype Query Language (AQL) queries [45].

Continuing, Ethercis—also known as Ethereal Clinical Information System—is a system, currently at the time of this writing in a beta mode, that allows simple interactions with web clients using a REST Application Programming Interface (API). It persists data in a separate database, supporting both RDBMS and NoSQL DBMSs. Its current version provides an interface for the PostgreSQL 9.4 DBMS. Queries are written in SQL and mixed with JSON functions and operators.

Other openEHR solutions include yourEHRM [46] by Atos Research that uses MongoDB as its internal DBMS, and an anonymous software package mentioned by Austin et al. [47] that uses PostgreSQL DBMS and forms the core of both an academic product named Cortext and a commercial one called HeliconHeart.

Finally, it is worth mentioning the work by Miranda Freire et al. [48] exploring the performance of NoSQL databases handling health care datasets up to 4.2 million records and comparing them to the conventional RDBMS MySQL. Specifically, in that work MySQL is compared to the NoSQL XML databases BaseX, eXist and Berkeley DB and to one document-oriented NoSQL database, Couchbase. With the larger datasets—which are most relevant to this work—Couchbase was found to outperform the other DBMSs in the tested single-node setup; no comparison were done in multi-node configuration.

## Materials and Methods

### OpenEHR

**Fig 4:**
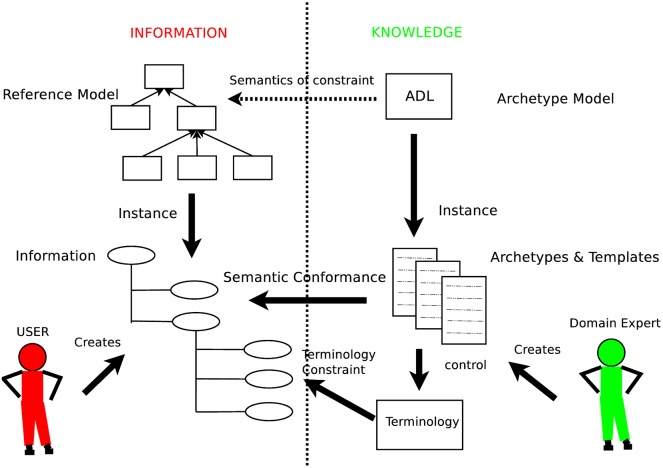
The openEHR two-level separation. Relationship between the Reference Model, on the left, and the Archetype Model, on the right.

OpenEHR is a standard evolved from the Australian Good Electronic Health Record (GEHR), whose most peculiar feature is the two-level framework, where the information model—also known as reference model (RM)—is kept separately from the clinical knowledge—that is the Archetype Model (AM)—see Fig 4. The information model defines the generic types and structures for record management. It is designed to be limited to domain-invariant data elements and structures, such as quantity, coded text and various generic containment structures—i.e., the classes. The AM provides, through archetypes and templates, structures and admissible values of a series of fields with well-defined semantic relationships. The archetypes are expressed using constraints on instances of the underlying reference model while the templates are models of content corresponding to use-case-specific datasets, composed of archetype elements.

Separating the two models has the advantage of separating the responsibilities in knowledge management and sharing. Medical professionals can author archetypes and IT programmers can develop the storage and sharing layer. The architecture is designed to optionally make use of external health terminologies, such as SNOMED CT, LOINC and ICDx. Each archetype is a computable definition—or specification—for a single, discrete clinical concept and is expressed in the ADL, which is defined by an ISO standard and can be viewed and reviewed in “clinician-friendly” formats—such as structured definitions and mind maps. Archetypes represent real world concepts, such as “patient”, “blood pressure”, or “antenatal examination”. Archetyped data have the same meaning regardless of the EHR context, the EHR system, and even regardless of the language. The ADL can be used to write archetypes for any domain that includes a formal object model to describe data instances. Archetypes are language-neutral, so they can be authored in and translated into any language. The ADL encloses three other syntaxes, cADL (constraint form of ADL), dADL (data definition form of ADL), and a version of first-order predicate logic (FOPL), to describe constraints on data which are instances of some information model.

**Fig 5:**
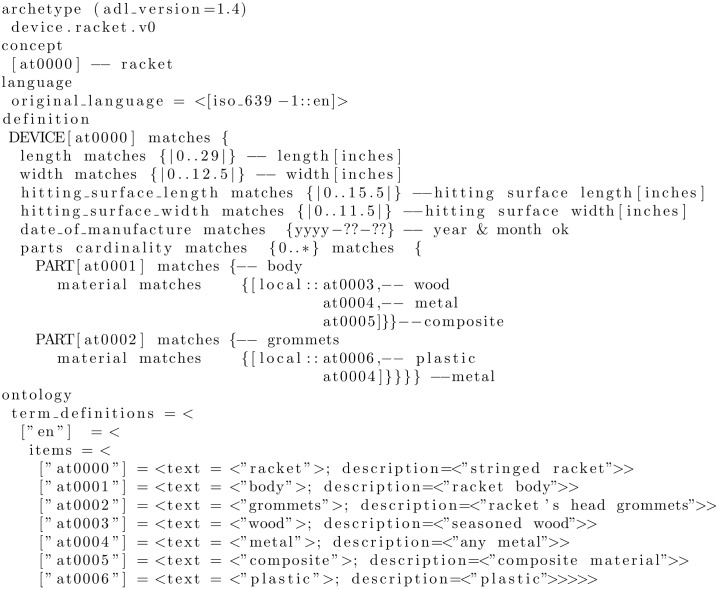
Example of an archetype expressed in ADL. The archetype defines a generic sports racket.

Fig 5 shows an example of a fabricated archetype. In the example, the idea of a sports racket is defined in terms of constraints on a generic model of the concept DEVICE. All sections are written in dADL except for the definition uses the cADL.

In ADL the main sections are called concept, language, definition and ontology. If the racket had been derived from another archetype then a specialize section would have been added to its concept section, akin to inheritance in programming languages. The definition contains the main formal definition of the archetype. It specifies the class and attributes created in the object model associated to this archetype—for instance, in Fig 5, class DEVICE and attributes length, width, etc. The attribute part has a constraint called cardinality, which is applicable to any attribute, and specifies the limits on the number of members instances of container types, such as lists and sets. In our example racket any number of instances of parts are allowed. Another important constraint, not illustrated in the example, is the ARCHETYPE_SLOT, which allows one archetype or a list of archetypes to be included within an archetype definition. That means that archetypes can be nested and combined to describe complex structures, such as the ones present in biomedical pipelines. Finally there is the ontology section, which defines the local nodes and constraints—elements whose names conventionally being with AT and AC, respectively—and how they are bound. In the latest release of ADL (version 2.0) this section has been renamed terminology.

It can be noted the repeated object/attribute hierarchical structure of an archetype that provides the basis for using paths to reference any node in an archetype, paths that follow a syntax subset of W3C Xpath. The data expressed as single archetypes or—more frequently—compositions of archetypes, can be queried in AQL. The AQL, formerly known as EHR Query Language (EQL), is a declarative query language born in 2005 to satisfy the following four requirements for an archetype-based query language [49]:

1. the query language should be able to express queries for any data item from an archetype-based system—i.e., data defined in archetypes and/or the underlying reference model;
2. the query language should be usable by both domain professionals and software developers;
3. the query language should be portable—i.e., neutral to system implementation, application environment and programming language;
4. the syntax should be neutral with respect to the reference model—i.e., the common data model of the information being queried. Particular queries will of course be specific to a reference model.

The main asset of AQL is that, unlike other query languages such as SQL or XQuery, it allows one to express queries at the archetype level, i.e., a semantic level different from the data instance level. This characteristic is the key to easily share queries across system boundaries or enterprise boundaries. Its main features are:

- openEHR archetype path syntax in the query expression;
- containment mechanisms to indicate the data hierarchy;
- ADL-like operator syntax, such as matches, exists, in, negation;
- a neutral expression syntax—AQL does not have any dependencies on the underlying RM of the archetypes. It is neutral to system implementation and environment;
- the support of queries with logical time-based data rollback.

**Fig 6:**
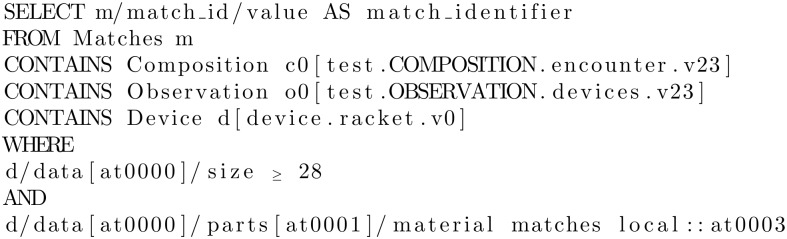
Example of an archetype query in AQL.

AQL has clauses similar to the well-known SQL, and in particular they are: SELECT, FROM, WHERE, ORDER BY, TIMEWINDOW. The SELECT clause specifies the data elements to be returned, using openEHR path syntax to indicate expected archetypes, elements, or data values. The FROM clause specifies the data source and the containment constraints introduced by the CONTAINS keyword. The WHERE clause defines, within the chosen source, data value criteria. The ORDER BY clause is used to select the data items that rule the returned result set order. Finally, the TIMEWINDOW clause restricts the query to the specified time or time interval. An example of an AQL query is given in Fig 6. The domain of sport matches “Matches” is searched for the ones where a wood racket with a length exceeding twenty-eight inches was used; if any exist, the query returns their match identifier.

The CONTAINS keywords allows one to navigate through the nested archetype. In particular, in the example the archetype racket must be inside a specified OBSERVATION archetype (*test.OBSERVATION.devices.v23*) which in turn must be inside a specified COMPOSITION archetype (*test.COMPOSITION.encounter.v23*). Between the two there may be any number of archetypes or no archetypes at all.

### Data Structures Indexing

OpenEHR provides a valid way of retain data semantics, context and provenance but something has to be done in order to search this data efficiently and in a scalable way.

Given the complex structure of the data, featuring deeply nested and repeated blocks, we decided to implement an indexing of unique structures to greatly ease the task of the database engine during queries.

**Fig 7:**
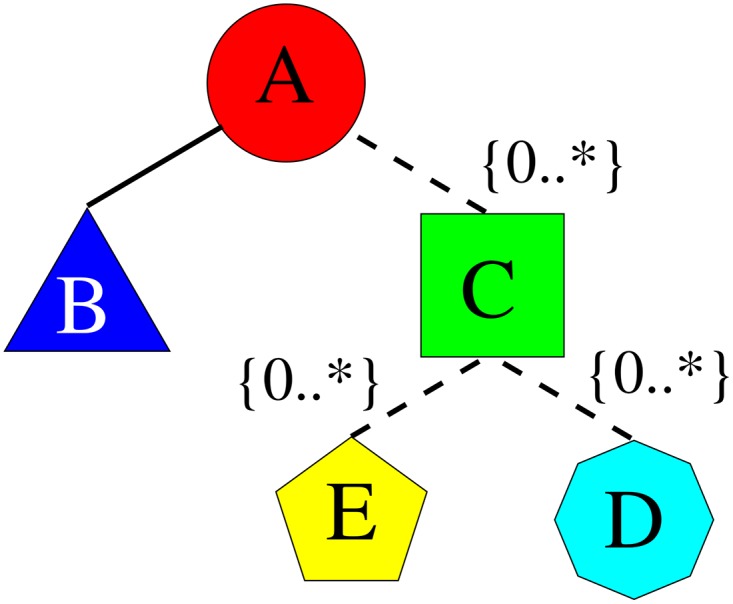
Example of an archetype, composed of other archetypes, shown as a tree structure. Different shapes/capital letters mean different archetypes. The text “0..*” shows the cardinality of the relation (i.e., from 0 to any number of the related archetypes).

As an example, Fig 7 shows an archetype that represents a class of structures.

**Fig 8:**
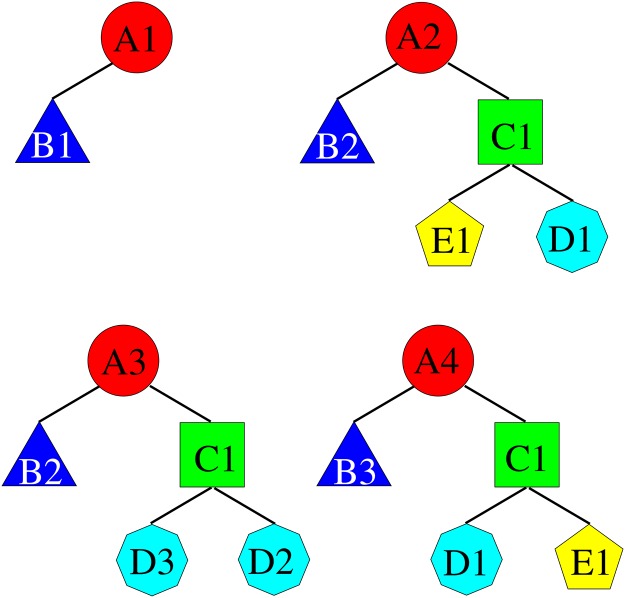
Some possible instances of the archetype illustrated in Fig 7. The number after the capital letter denotes a particular instance of the given archetype.

In the structure each node, denoted by a shape and a capital letter, is also an archetype. Different shapes/letters mean different archetypes while the text “0..*” indicates the cardinality of the relation (i.e., there can be from 0 to any number of the related archetypes). On the other hand, Fig 8 shows some structures that are instances of the archetype in Fig 7. The number inside the shaped node, next to the capital letter, indicates a given instantiation. We have instances with no octagon nodes and instances with one or two archetypes of that type. This structure is very difficult to map to the conventional relational DBMS, since it involves schema changes, thus any implementation in such databases would be temporary and fragile. Regardless of the specific DBMS used, indexing the structures saves time while traversing the data tree looking for query matches.

**Fig 9:**
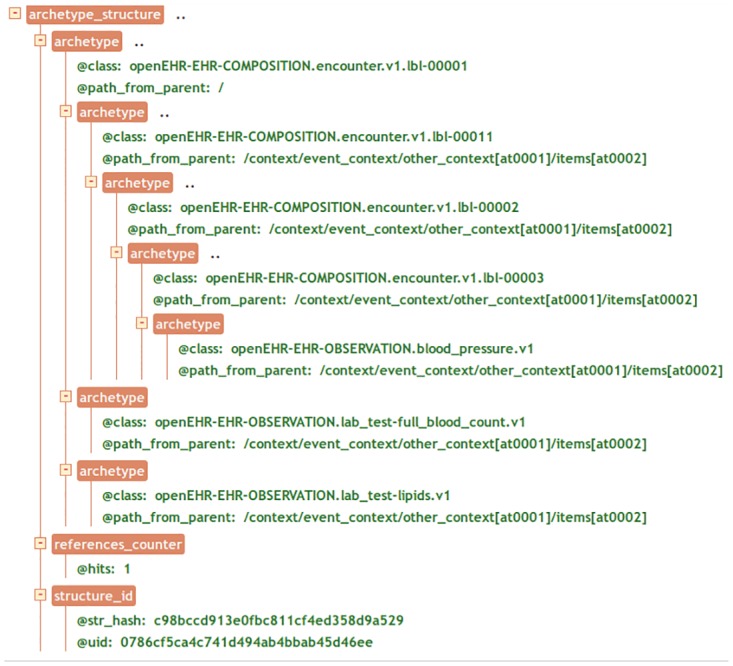
Tree view of an archetype data structure with multiple nested archetypes.

In practice, to index our structures each one is is pruned of non–structural details, analyzed for the mutual position of archetypes, and transformed into a canonical description. The latter is given an identifier and stored in a specialized database. This is one of the responsibilities of the Index Service. Archetypes that occupy different places in a list of items—i.e., permutations—are assigned the same structure identifier, avoiding overloading the Index Service with duplicate structures. Fig 9 shows an example of a structure that contains six archetypes differently nested inside the containing archetype *openEHR-EHR-COMPOSITION.encounter.v1.lbl-00001*.

The class property defines the archetype name whereas the path_from_parent property indicates a path relative to the closest containing archetype. All archetypes in the example, except for the root archetype, have the same path_from_parent property and the reason is that they refer to the same type of archetype, a *openEHR-EHR-COMPOSITION.encounter*, whose path to the contained archetype is always the same.

**Fig 10:**
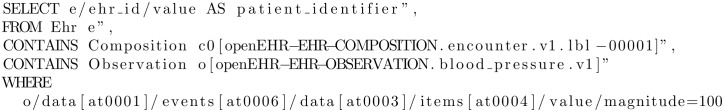
AQL query for the data structure in Fig 9.

During the query process the Index Service is asked to perform another task. Let us suppose to have to evaluate the AQL query shown in Fig 10 on the previously defined structure of Fig 9.

The Index Service looks for all the structures that match the containment constraints and returns a list of their ids along with the absolute path or paths to get to the innermost archetype of the CONTAINS clause. For example, the structure in Fig 9 matches the containment constraint in the query, so the unique identifier of the structure and the path are returned by the Index Service. In this case, there is only one match; its path is computed by concatenating all the archetypes’ relative paths from the root to the last contained archetypes. Thus the Query Management System, that will be described in the next section, has all the information to search for the instances that match the WHERE clause while examining only a subset of all the stored records—i.e., the ones whose structure satisfies the containment clauses.

### Implementation

PyEHR is an open source data access layer designed to help build applications for secondary use of clinical and biomedical data. The software is written mostly in Python, and exposes its services through a REST interface Its goal, as already mentioned, is to efficiently handle complex heterogeneous structured data. The system implements the openEHR standard, storing archetype representations and evaluating AQL queries; it also uses advanced data indexing to reduce query processing time, especially on large datasets. These features give it flexibility both in terms of accepting and querying complex data structures.

**Fig 11:**
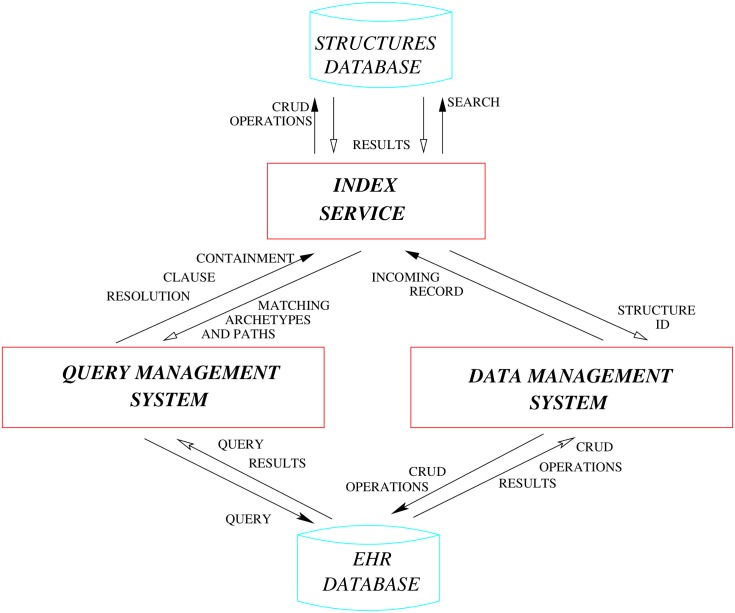
PyEHR architecture: main modules, databases and their interactions.

The overall architecture of PyEHR is summarized in Fig 11. There are three main modules: the Data Management System, the Query Management System and the Index Service. These modules interact with each other and with two databases: the EHR Database and the Structures Database. The Structures Database stores unique data structures. The EHR Database, which is interchangeable, stores biomedical data and other metadata, including structure identifiers, that link the data and the Structures Database. Finally the Index Service is implemented using BaseX [50], which is used as a reference database for managing and querying index structures.

**Fig 12:**
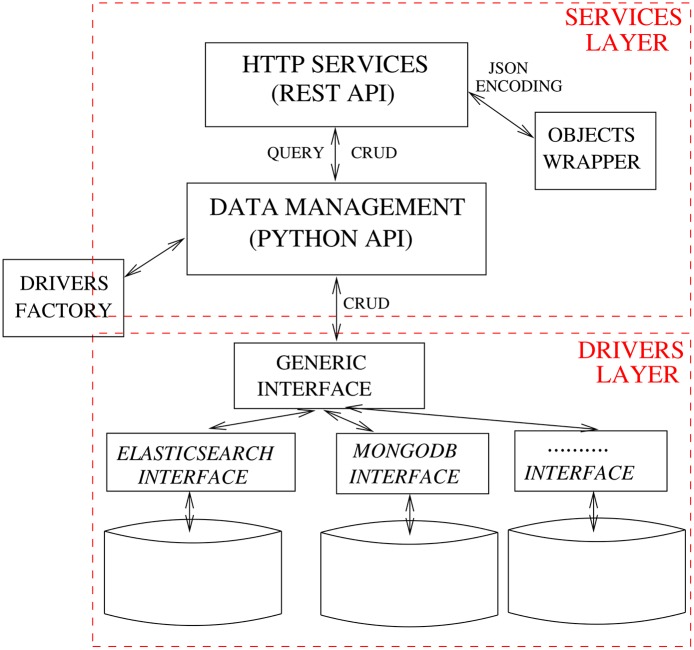
PyEHR architecture: Data Management System.

The Data Management System handles data storage and retrieval to and from the chosen EHR Database. The module is formed by two layers: a service-oriented API for managing the data, and a driver interface that supports multiple database back-ends (Fig 12). The biomedical data, though expressed according to a standard—i.e., openEHR—can be stored in the chosen database in a variety of formats; for instance, Entity-Attribute-Value [51] is a good choice for table-based storage systems, XML is the natural choice for XML databases [52] and JSON, YAML and XML are all well suited for document-oriented databases [53]. In PyEHR the choice of the format is left to the developer of the specific database driver plugin, allowing him/her to select the format that best suits the specific database. Every time a new record is inserted, the Data Management System forwards it to the Index Service. There, its structure is normalized and compared to the ones already in the Structures Database; if the structure is new it is added. The identifier of the structure is returned to the Data Management System, which stores it along with the encoded record into the EHR Database.

**Fig 13:**
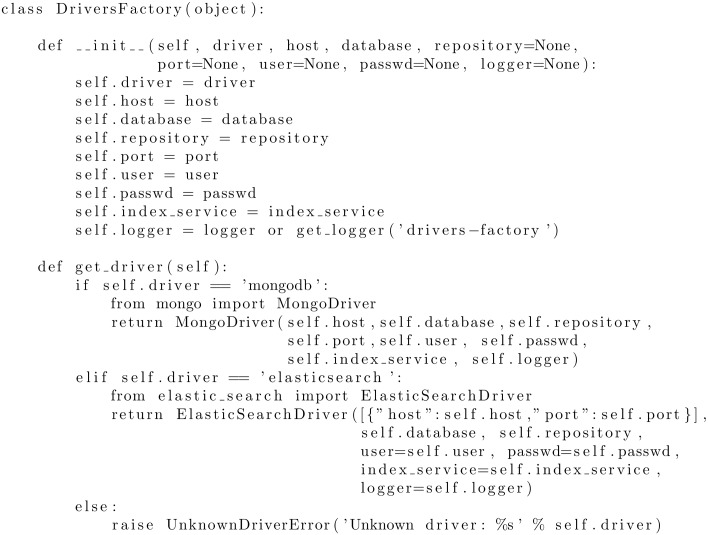
Code snippet from the Driver Factory Class. The actual driver factory class; the lines at the bottom refer to the drivers for the MongoDB and Elasticsearch databases.

A key element of the Data Management System is the driver interface, that is designed to easily allow swapping the underlying DBMS, analogously to strategies employed in previous work [54], In fact, implementing a new database driver only requires the addition of an entry in the driver’s factory class (see listing in Fig 13) and the implementation of the short list of routines in the driver API. At the time of this writing, PyEHR supports two NoSQL DBMS: MongoDB and ElasticSearch. Both systems, having been designed to handle hierarchical sets of key-value items and are easily adaptable to the document-like structures of openEHR data.

**Fig 14:**
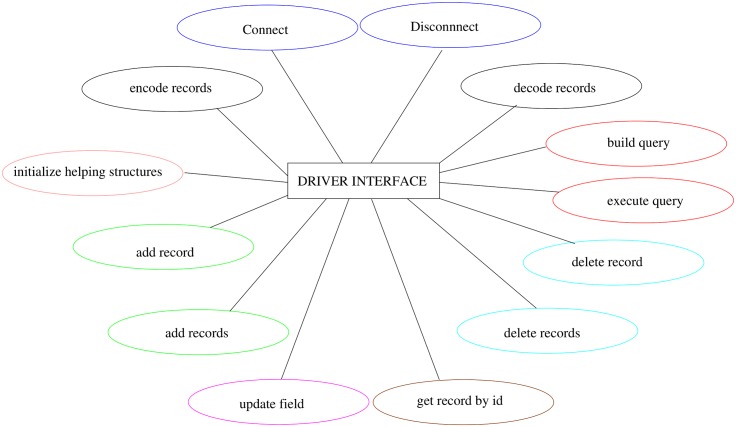
Main driver API tasks.

Fig 14 shows the main tasks performed through the driver API. For clarity, the functions pertaining to document versioning have been omitted.

Each driver interface has the following responsibilities:

- manage connections to and and disconnections from the EHR Database;
- implement Create, Read, Update and Delete (CRUD) data operations;
- handle queries (translating them to the database’s natural query language and executing them);
- encode/decode data to/from the wrapper objects defined in the services layer, automatically converting any special characters;
- create data structures such as SQL tables or folders;
- split or join records as required by the underlying storage system.

In some cases, the driver interface may have to work around limitations or incompatible features in the database management system. For instance, Elasticsearch does not allow attributes with the same name but different class under the same “index–type” combination—e.g., foo defined as an integer in one document and as a dictionary in another. To overcome this limitation, the Elasticsearch driver in PyEHR changes the type name, creating a unique one by associating a generic name to the structure identifier. This solution requires the creation of a lookup table to quickly retrieve the “index–type” couple information for a given document identifier; this event occurs quite frequently, especially when managing document versioning. The service layer has its own representation of data objects so the driver also acts as a translation interface that converts to and from the well-known JSON format. This strategy allows the data objects to be equally handled by the REST API and the database engine even though their data representation may differ. For instance, clinical records that are represented internally as documents, whose structure closely matches the original, written in ADL; when needed these are converted to a JSON representation and passed in this format to the database driver or to the REST API.

**Fig 15:**
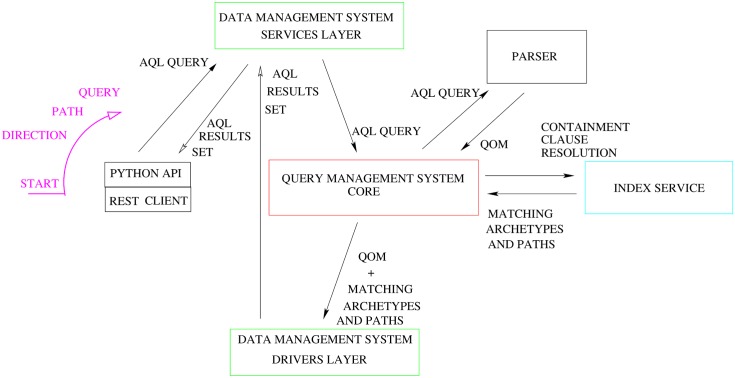
PyEHR: Query flow. The query evaluation process starts from nine o’ clock and proceeds clockwise.

The Query Management System directs and interacts with the different components to evaluate queries. Fig 15 shows the steps followed by the system as it processes a query, starting at nine o’ clock and going clockwise. The first step involves the input acquisition. The AQL input query is submitted through the REST interface, leveraging the python API, through command line interface or some other program. The Service Layer of the Data Management System receives the query and forwards it to the Query Manager Service Core (QMSC), which instantiates a Parser to process it. The Parser converts the AQL query string into a Query Object Model (QOM) instance, which has access to the query’s data and structure via API calls. The location part of the QOM, which has the CONTAINS clause (see section on queries creation), is then given to the Index Service. A list of matching structures and associated paths is returned to the QMSC, which forwards them along with the QOM to the Driver Layer of the Data Management System. The Driver Layer translates the QOM into the database-specific query, executes it and returns the results as an AQL Results Set—a simple format that represents query results as sets of columns and rows. The results are returned to the user through the Service Layer of the Data Management System.

### Scalability Assessment Preparation

#### Artificial Data Creation

For the purpose of evaluating the performance of PyEHR we created synthetic data that are similar to real data and at least as challenging, if not more, for the query system. The generated data effectively tests the worst-case scenario. Two test were conducted: “Constant Number of Records” and “Constant Load”. For the “Constant Number of Records” test, we generated ten million EHRs, five million of which are “filler”, not responding to any of our test queries, and five million comprise five one-million-record dataset—each responding to its respective queries. The reason for the five datasets is to check the sensitivity of query time to the position of the matching records; each dataset answer to the same type of query with the identical amount of records but these records appear in different places of the structures. For the “Constant Load” test we chose a straightforward approach, generating a ten-million-record dataset step by step. We created a ten percent of records responding to queries along with ninety percent of “filler” and replicated the process ten times.

Each EHR is written using the openEHR formalism—i.e., ADL—and is composed of different archetypes nested to form a tree type structure.

**Fig 16:**
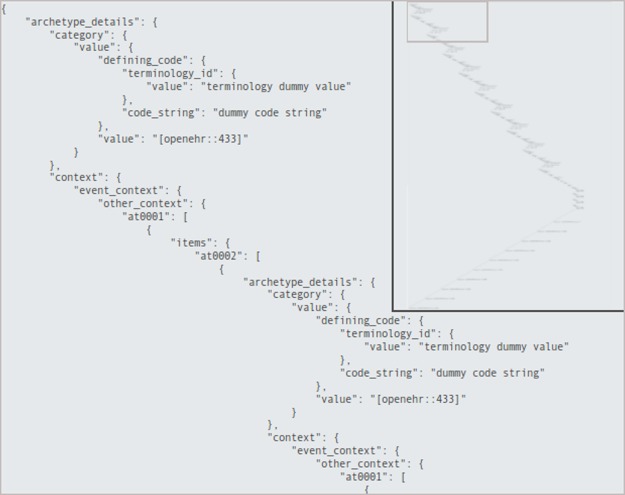
JSON view of a generic EHR structure. The part on the left is the magnified view of the small region of the whole structure, shown on the top right.

We created a set of complex and varied EHR structures to simulate, through aggregations of archetypes, the output of biomedical pipelines. Each EHR structure has the appearance –coded in JSON—of Fig 16. Each record is generated by sampling randomly from this set of base structures and instantiating a new record with new values, as to obtain the desired number of matches to the queries.

**Fig 17:**
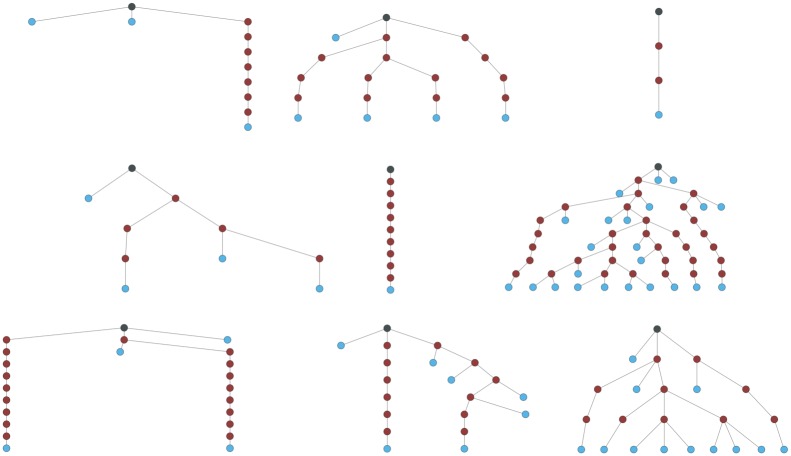
Graph representation of some EHR structures generated. Archetypes are shown as circles in a tree structure.

We built and instantiated about 2600 unique structures. Fig 17 shows a small sample. The circles in the diagrams represent the archetypes; the deepness of the structures should be apparent from the illustration.

**Fig 18:**
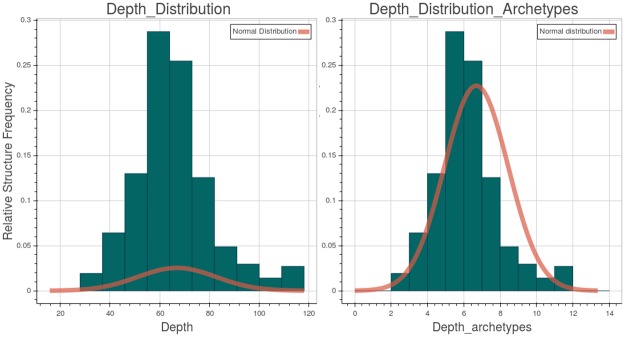
Structures relative depth distribution on the created EHRs. On the right the archetype maximum depth vs the relative structure frequency. On the left the overall maximum depth vs the relative structure frequency.

To quantify the characteristics of the test structures, Fig 18 depicts various metrics. Respectively, from left to right, the Figure shows: the maximum relative depth distributions of all the JSON elements, including both the archetypes and the archetypes’ contained elements; the maximum relative depth distribution of the archetypes. A normal distribution, with the same mean and standard deviation of the underlying data, is drawn over each diagram. From the statistical point of view, the structures have a mean “maximum depth” of about 6.7 for the right graph with a standard deviation of 1.7, and a mean of about 66 and a standard deviation of 15.7 for the left one, that is each archetype has roughly a mean maximum depth of 10. The complexity can be high as there are structures that can reach over 110 nested elements and contain up to 12 archetypes in a single branch direction. The maximum depth in the archetype distribution is very similar to the normal distribution.

**Fig 19:**
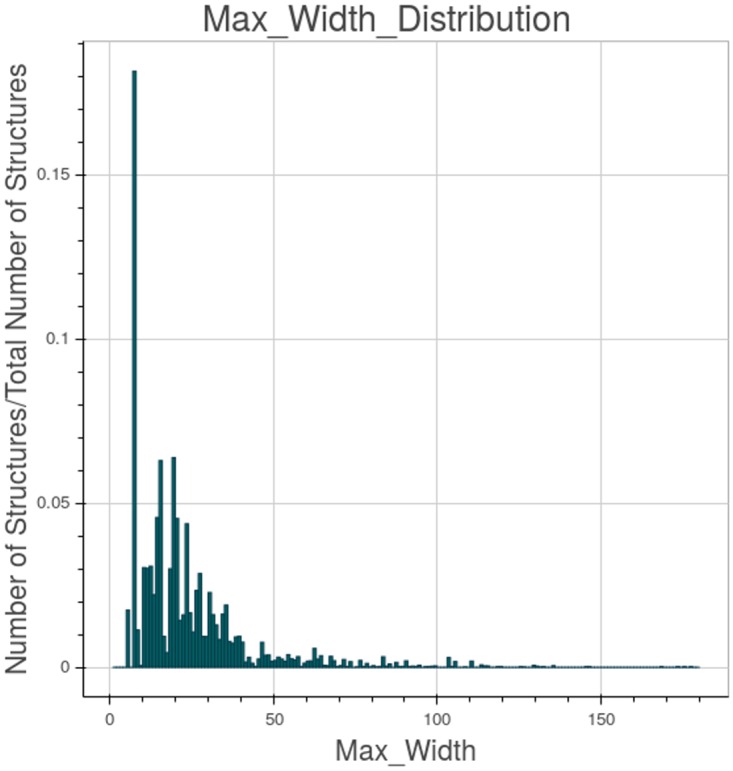
Structures “max width” distribution on the created EHRs.

The maximum width is represented in Fig 19. The width is the number of elements measured at each level of the tree structure. The modal value for its maximum is 8 but it can reach a value greater than 150.

#### Query Creation

**Fig 20:**
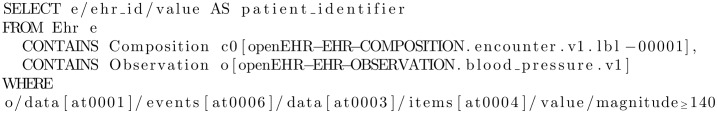
Type 3 query example with two levels of containment. The number of CONTAINS clauses gives away the query level.

We created five types of AQL queries, to which we assign a number and a brief name for future reference. They are, in ascending complexity: “1-match single”, “2-match multi”, “3-hit single where”, “4-hit multi and where”, “5-hit multi or where”. The “1-match single” query type has a single element in both the SELECT and FROM clauses. The “2-match multi” query type has two elements to be returned in the SELECT clause and a single FROM clause. The “3-hit single where” query type has a single element in the SELECT, FROM and WHERE clauses. The “4-hit multi and where” query type differs from the third type only for the existence of a second WHERE clause joined with an AND condition to the first one. Finally, the “5-hit multi or where” query type has, with respect to the fourth type, the two WHERE clauses in OR. All FROM clauses have one or more CONTAINS keywords, because of the nested nature of the data structures; this feature adds to the complexity of the query. Each query is performed on 4 different levels of containment—i.e., number of CONTAINS. For instance, Fig 20 shows two levels of containment for a type 3 query.

In the example, an EHR matches the given query if it has, anywhere within its structure, the blood pressure observation archetype *openEHR-EHR-OBSERVATION.blood_pressure.v1* within a composition archetype *openEHR-EHR-COMPOSITION.encounter.v1.lbl-00001*. Between the two archetypes there may be any number of archetypes, included none. Depending on the number of results we let each query yield a simple count of the matching EHRs or both a count and the matching records. The threshold for fetching the results has been set to 10k records, hence under that value the records are counted and returned. The first two types of queries have a number of results that goes from 175k records, at level 5, to 1M records, at level 2, so only a count is performed with them. The last three types of query range from 1.75k, at level 5, to 10k, at level 2, so both count and fetch phases are executed. It should be noted that the records that match a query at a given level also match the query at any higher level.

#### Baseline Comparison Reference

A comparison reference is dictated by the need to understand whether the measured system performance is competitive with existing solutions. Among the full spectrum of the solutions to our problem, we can imagine two diametrically opposite approaches. The first one is to use a relational database management system to store the data. The difficulty encountered by this approach lies in retaining the relationships between entities. This requirement calls for a very complex arrangement of tables—assuming it exists; it would be an ad hoc solution with a delicate balance that any modification, addition or removal of data or relationships may easily break. In short, this approach incurs high maintenance costs. The second approach is a sort of brute force approach. It implies the use of a big data search and query platform, therefore designed to be scalable. For our baseline solution, we chose the latter approach, which is the safer and simpler of the two. We adopted Apache Hadoop MapReduce, one of the most well-known big data frameworks for writing scalable applications. This framework has already been applied to processing big data in the medical sector [55–58]. The data, once loaded onto the Hadoop Distributed File System (HDFS), is analyzed by a recursive search algorithm to find the records that match the given query. We restricted the comparison to the most simple queries—the type 1 (match single) queries—with all their different levels of containment.

#### Data Insertion

The data insertion performance of the two NoSQL databases used in this work, and in particular with Elasticsearch, has shown to be very sensitive to parameter tuning. However, since this work is focused on query performance, we did not concern ourselves with optimizing it. Nevertheless, in a production application this issue would certainly deserve significant attention. The time required to insert our ten-million-record dataset, from scratch, into the various data stores is roughly as follows: about two days for MongoDB; about four days for Elasticsearch; for HDFS, merely the time to copy the data to disk using Apache Hadoop’s distcp, which is, with the cluster decribed in the “Constant Load” results section, about thirty minutes.

## Results

### Constant Number of Records

The “Constant Number of Records” test (CNR for short) is, as the name suggests, a test where the number of records remainst constant while varying the number of working nodes. We ran the test on Amazon Web Services (AWS) using the instance model “r3.2xlarge”, which is one of the “Memory Optimized Instances” offered by the service. Each node has 8 virtual High Frequency Intel Xeon E5-2670 v2 Ivy Bridge 2.5GHz Processors, 61 GiB of RAM and one SSD disk, 160 GB in size

For the test, we configured Apache Hadoop YARN and HDFS to run on twelve nodes. Ten nodes are configured as workers, while two are used as head nodes running the various management services for YARN and HDFS. It has to be underlined that ten nodes have always been committed to the storage in HDFS, for replication necessity, even when fewer than ten nodes were used for the calculations. In PyEHR calculations the two control nodes are used for the main program, i.e., pyehr, and for the database master. The other ten are used as slaves by the database manager system to store, query and retrieve the data. In contrast with Apache Hadoop MapReduce, the number of nodes that actually store the data assumes any value from one to ten as we add nodes.

To ensure a fair comparison to the baseline, before choosing the final hardware type many tests were performed to evaluate the impact of disk, CPU, network bandwidth and memory size on query time. We ran tests varying the number of disks (from one to twelve), the type of disks (SSD vs HDD), the network bandwidth (1Gbps, 10Gbps), the amount of memory (from 16 GiB up to 121 GiB) and the number of processors (from 8 to 20). These experiments showed that the processing on Apache Hadoop MapReduce—for this specific problem—is mainly cpu bound, given a sufficient amount of RAM, so we opted for an AWS instance type that provides a good compromise between these requirements and the desire to consider only commodity hardware.

The following subsections present the results of our experiments. The results show the effect on the query total elapsed time of both the query type variation and the query containment level variation in addition to the time cost of the index lookup, count and fetch phases. Moreover, the “spread” graphs measure the results’ sensitivity to the position of the matching records in the dataset.

In all the figures, the mean value of the calculations is presented as a point, while a bar is used for their standard deviation. All tests were repeated 10 times. However, except for the “spread” graphs, all five datasets have been used to enrich the statistics thus mean value and standard deviation generally refer to 50 calculations. In the “spread” graphs we used only ten calculation to compute mean and standard deviation. Further, most of the results show the record count time rather than the record fetch time. The reason for this choice is that, though fetching is often required in real applications, it adds to the count operation only a trivial data upload operation. Moreover between counting and fetching the former operation is the most challenging with respect to the comparison, because Apache Hadoop MapReduce performed it very efficiently in its reducer phase.

#### Apache Hadoop MapReduce

**Fig 21:**
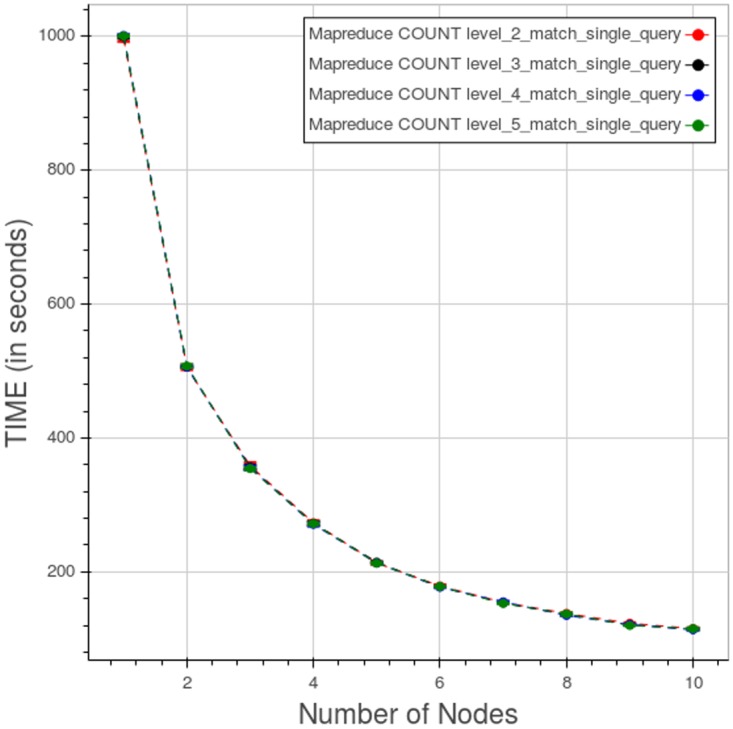
Apache Hadoop MapReduce, CNR. Query time vs number of nodes for a type 1 count query at different levels. Each point is the mean query time, while the bar shows the standard deviation of the measurements. At the scale shown the points and the bars are almost indistinguishable.

Fig 21 shows the behaviour of Apache Hadoop MapReduce for a type 1 count query. The curves for the four different levels are contained within few seconds. The maximum query time, associated to the single node cluster, is about 1000 seconds while the minimum, associated to the ten-node cluster, is about 100 seconds.

**Fig 22:**
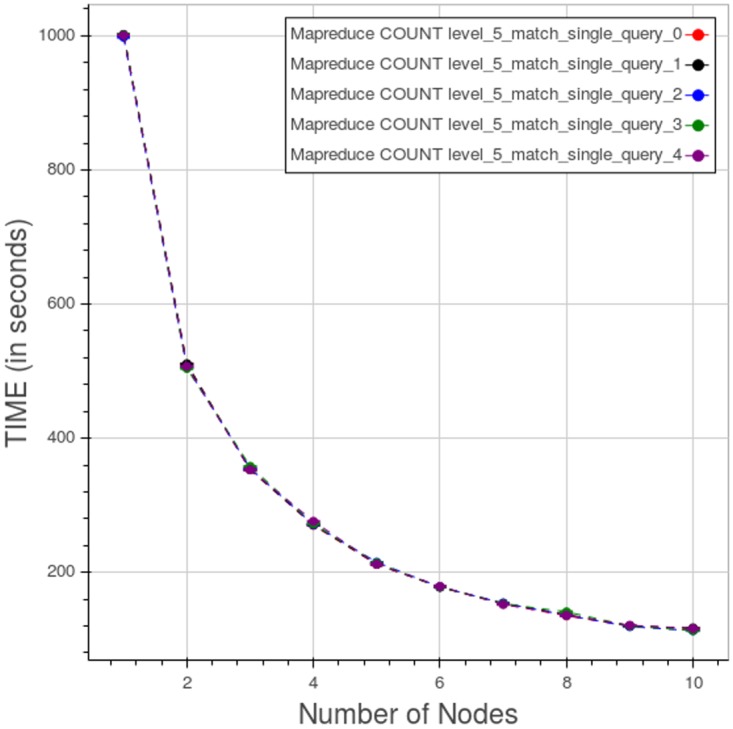
Apache Hadoop MapReduce, CNR. “Spread” curves for a type 1 count query at level 5. Each point is the mean query time, while the bar shows the standard deviation of the measurements. At the scale shown the points and the bars are almost indistinguishable.

The “spread” curves for a type 1 count query at level 5, obtained considering separately the five datasets, are displayed in Fig 22. The curves are very close; the larger difference between the curves is about four seconds.

#### PyEHR with MongoDB

**Fig 23:**
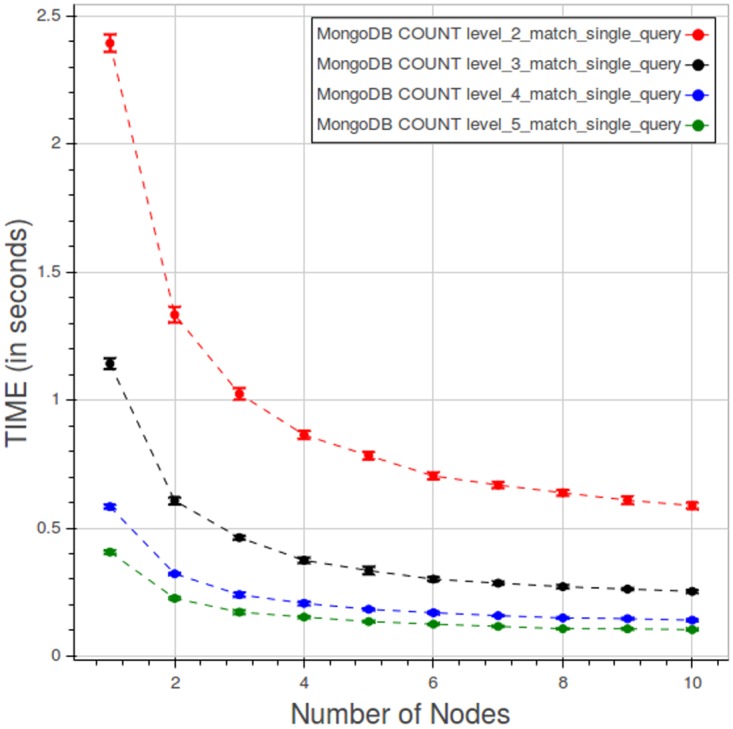
PyEHR with MongoDB, CNR. Query time vs number of nodes for a type 1 count query at different levels. Each point is the mean query time, while the bar shows the standard deviation of the measurements.

Fig 23 shows the performance of PyEHR with MongoDB for a type 1 count query at the four different levels. The low level queries take much more time because they involve a greater number of records.

**Fig 24:**
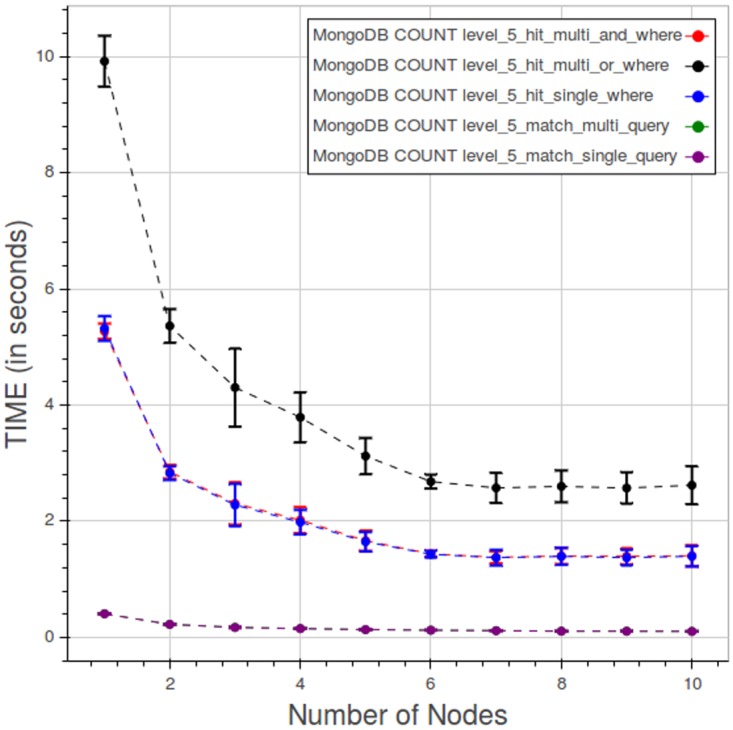
PyEHR with MongoDB, CNR. Query time vs number of nodes for different types of count query at level 5. Each point is the mean query time, while the bar shows the standard deviation of the measurements.

Fig 24 shows the behaviour at level 5 when varying the types of query. The query of type 5 with two WHERE clauses in OR is the most time consuming, in our tests, while the query of type 4 with two WHERE in AND and the type 3 with one WHERE are very close to each other. The queries without WHERE are clearly faster than the rest.

**Fig 25:**
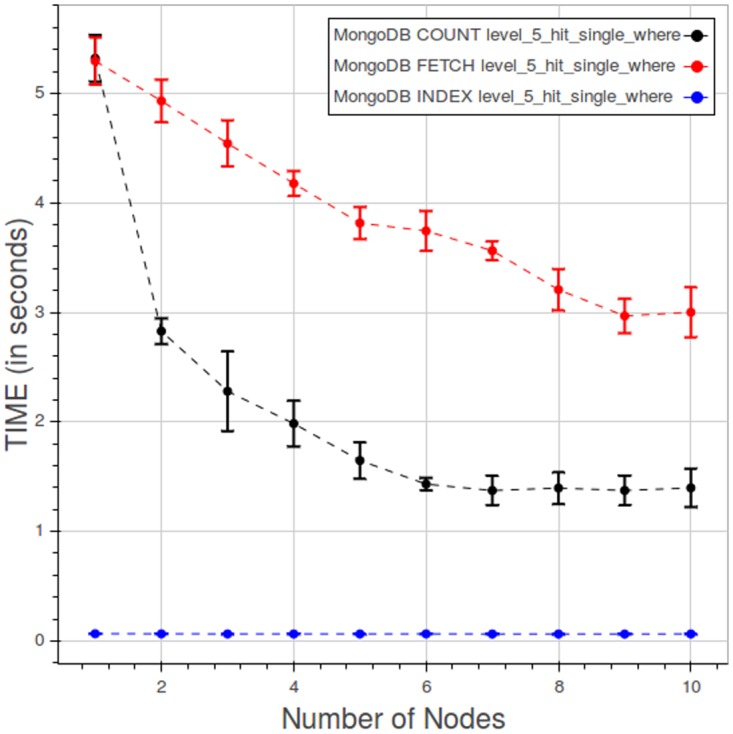
PyEHR with MongoDB, CNR. Query time spent in the index lookup, count and fetch phases while performing a type 3 query at level 5. Each point is the mean query time, while the bar shows the standard deviation of the measurements.

Fig 25 indicates how, in a query of type 3, the elapsed time is divided among the index lookup, count and fetch phases. Index lookup consumes a very little, close to constant, amount of time. Time spent in the count and fetch phases improves rapidly as we add nodes, up to five nodes; beyond five nodes the curves flatten as the improvement diminish.

**Fig 26:**
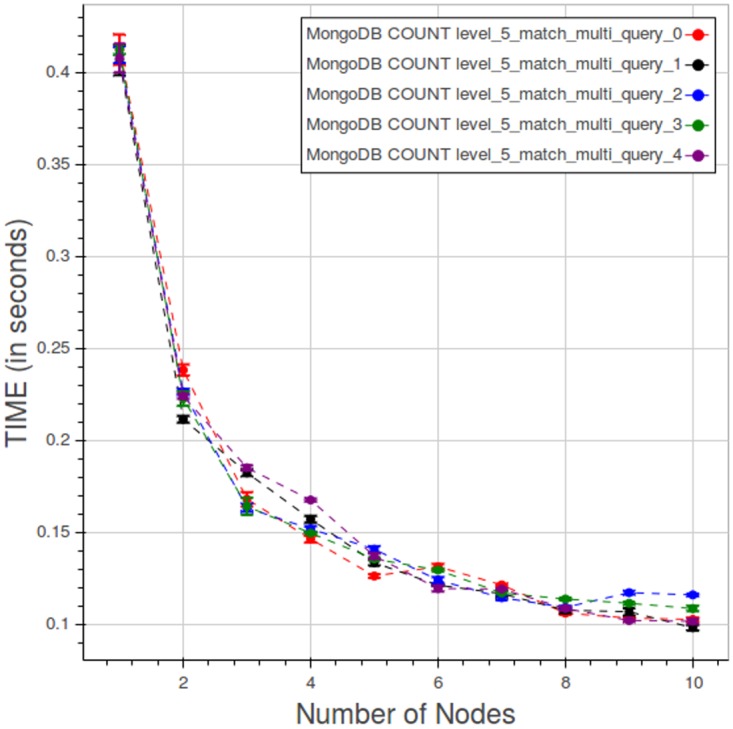
PyEHR with MongoDB, CNR. “Spread” curves for a type 2 count query at level 5. Each point is the mean query time, while the bar shows the standard deviation of the measurements.

Fig 26 shows the sensitivity of results for a type 2 count query at level 5 to the position of matching data in the dataset. The curves are quite close.

#### PyEHR with Elasticsearch

**Fig 27:**
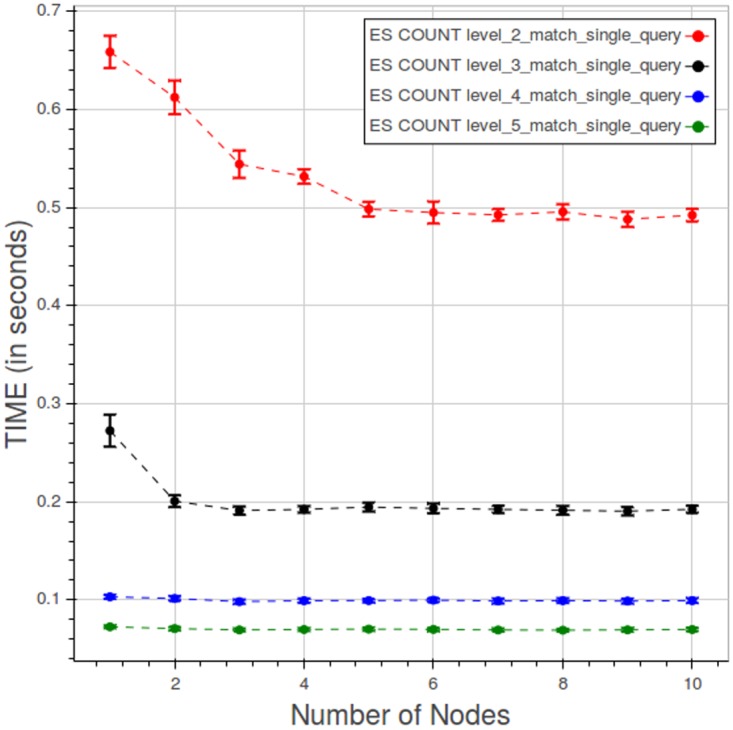
PyEHR with Elasticsearch, CNR. Query time vs number of nodes for a type 1 count query at different levels. Each point is the mean query time, while the bar shows the standard deviation of the measurements.

The behavior of PyEHR with Elasticsearch for a type 1 count query at different levels is illustrated in Fig 27. The scale of the chart is very small with respect to the runs with MongoDB and even more compared to the Apache Hadoop MapReduce graph. However, as with MongoDB, query time falls as we go up with the levels. For any given level, the results improve up to a certain point, depending on the level, and then the curve flattens.

**Fig 28:**
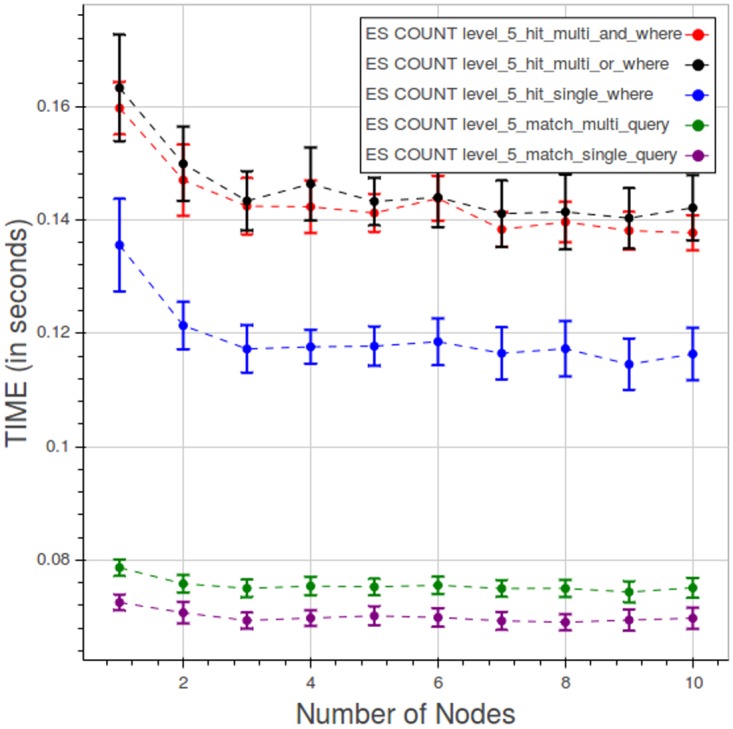
PyEHR with Elasticsearch, CNR. Query time vs number of nodes for different types of count query at level 5. Each point is the mean query time, while the bar shows the standard deviation of the measurements.

Fig 28 shows the curves for the five types of count query at level 5. Note that the scale used in this graph is smaller than the previous ones. We can see two clusters of curves, one for type 1 and type 2 queries ad the other for the remaining ones.

**Fig 29:**
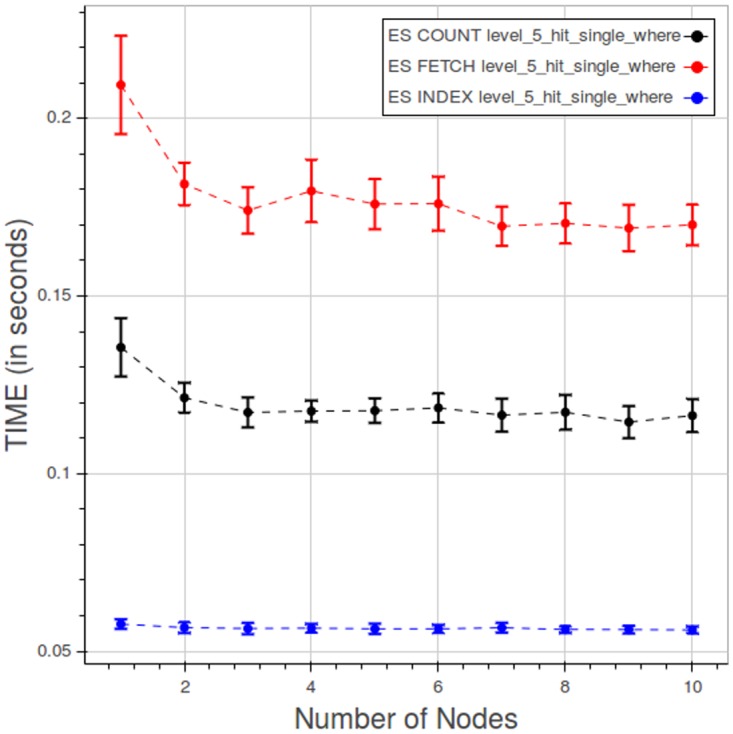
PyEHR with Elasticsearch, CNR. Query time spent in the index lookup, count and fetch phases in a type 3 query at level 5. Each point is the mean query time, while the bar shows the standard deviation of the measurements.

The portions of query evaluation time spent in index lookup, count and fetch phases are shown in Fig 29. As expected the fetching takes much more time than the other two operations. In this case index lookup time cannot be neglected, due to the lower overall operation time.

**Fig 30:**
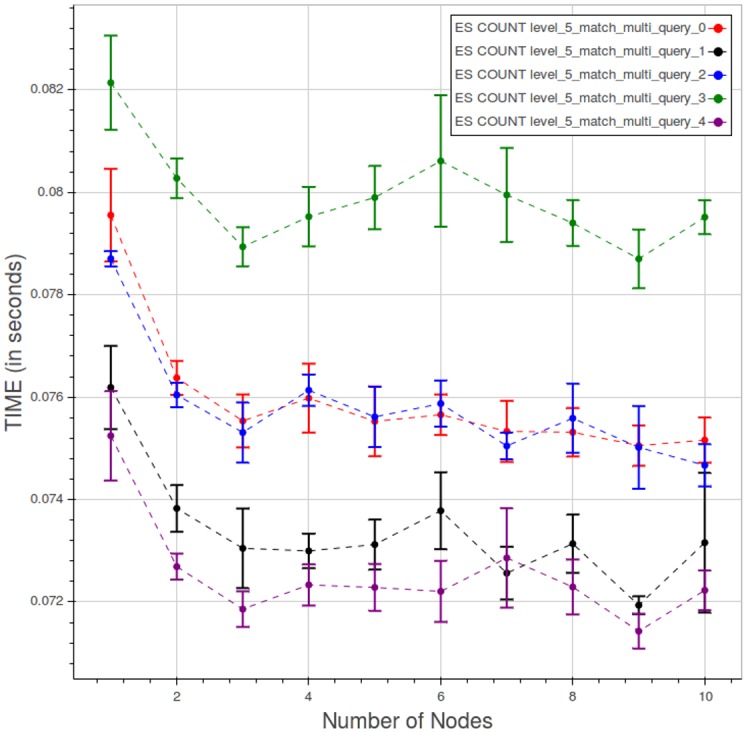
PyEHR with Elasticsearch, CNR. “Spread” curves for a type 2 count query at level 5. Each point is the mean query time, while the bar shows the standard deviation of the measurements.

Fig 30 shows the sensitivity of results for a type 2 count query at level 5 to the position of matching data in the dataset. The curves are closely gathered, with a maximum separation between the farthest datasets of about 9 thousandths of a second.

### Constant Load

The “Constant Load” test (CL for short) demands a concurrent linear change in both the number of records and the nodes, maintaining constant the load per machine, hence the name. It was performed on a local private cluster at the CRS4 research center. The nodes in the private cluster are equipped with two Intel Xeon E5440 2.83GHz (4 hyperthreaded cores each), 16GB of RAM and one HDD 240GB in size. Just like for the CNR test, twelve nodes were used: ten worker nodes for storing data and performing the actual computations and two head nodes for YARN/HDFS or pyEHR/MongoDB/Elasticsearch management services and launching queries. Each run was repeated five times. As in the previous case, most of the graphs presented show the count results.

#### Apache Hadoop MapReduce

**Fig 31:**
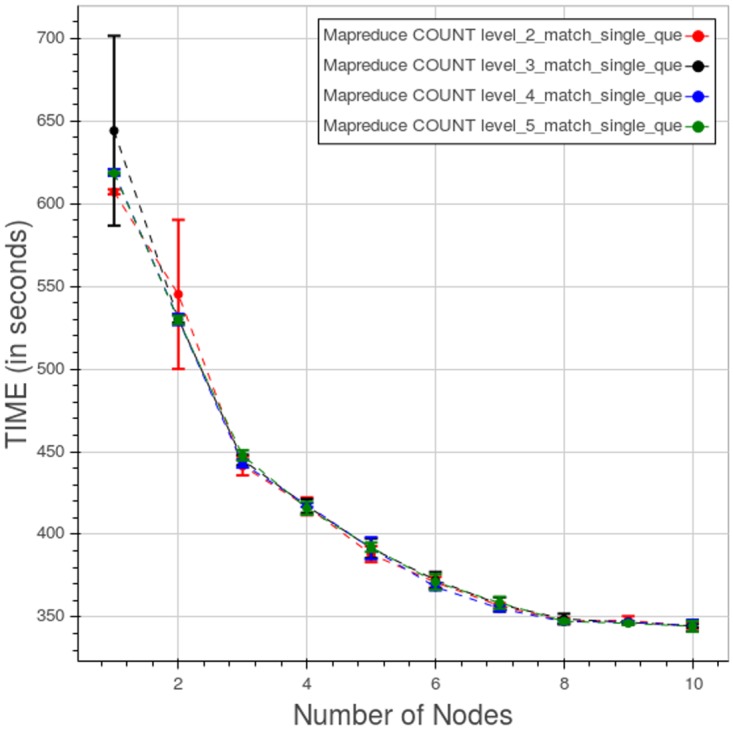
Apache Hadoop MapReduce, CL. Query time for type 1 count query at different levels. Each point is the mean query time, while the bar shows the standard deviation of the measurements.

Fig 31 shows the performance of Apache Hadoop MapReduce for a type 1 query at different levels. The points are relatively close, except for the first two nodes where the distances between the levels and the standard deviations for each level are large.

#### PyEHR with MongoDB

**Fig 32:**
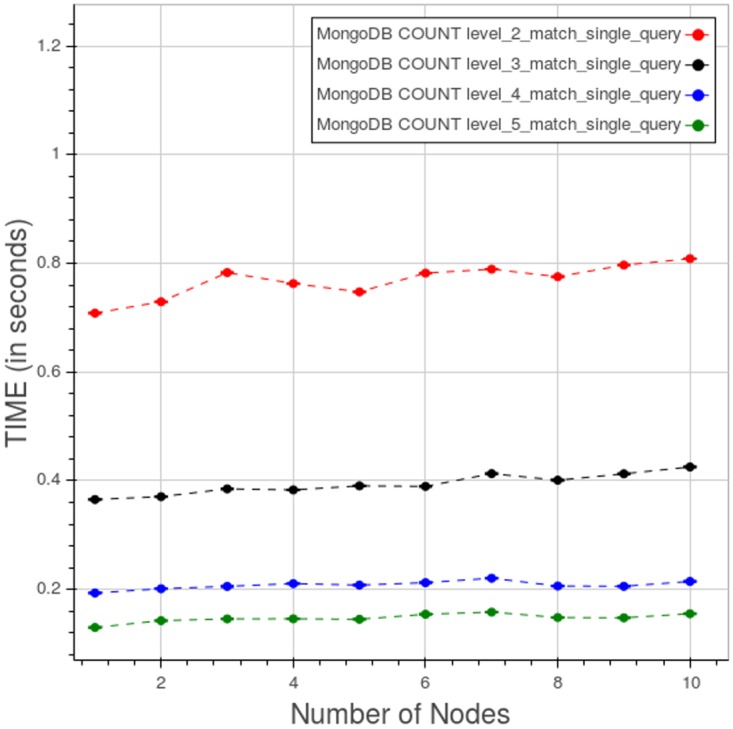
PyEHR with MongoDB, CL. Query time for type 1 count query at different levels. Each point is the mean query time, while the bar shows the standard deviation of the measurements.

Fig 32 shows the behaviour of PyEHR with MongoDB for a type 1 query at the four different levels. The curves are fully separated.

**Fig 33:**
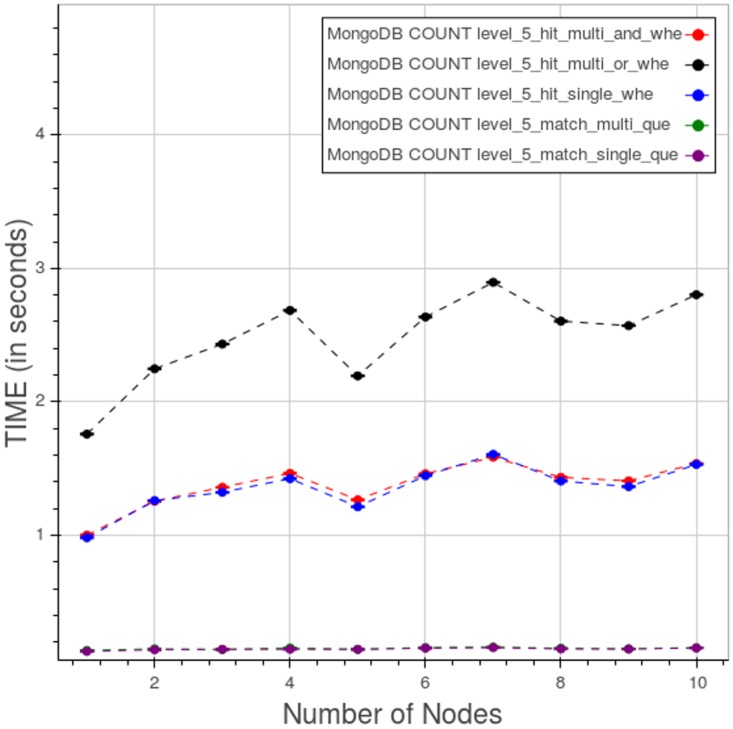
PyEHR with MongoDB, CL. Query time for different type of count queries at level 5. Each point is the mean query time, while the bar shows the standard deviation of the measurements.

The effect of changing the type of query at level 5 is shown in Fig 33. The type 3 query, with a single WHERE clause, and the type 4 query, with two WHERE clauses in AND, are very close; so are the type 1 query, with a single SELECT clause, and the type 2 query, with multiple SELECT clauses. There are anomalies at the nodes 5 and 7 that perturb the last three types of query. That was due to a non-exclusive use of those two nodes. We decided not to repeat the calculations since the curves behaviour is already established.

**Fig 34:**
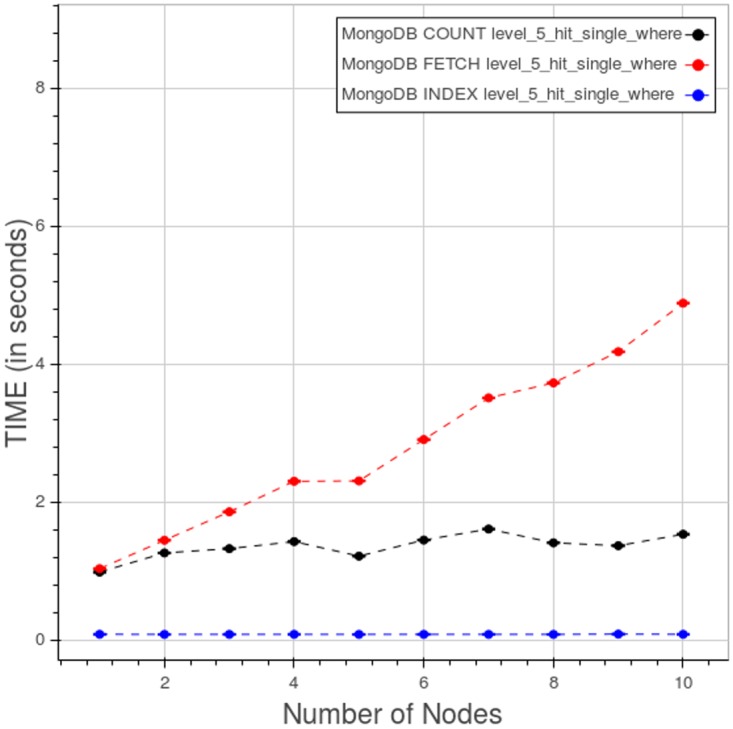
PyEHR with MongoDB, CL. Query time spent in the index lookup, count and fetch phases in a type 3 query at level 5. Each point is the mean query time, while the bar shows the standard deviation of the measurements.

Fig 34 shows the portions of query time ascribed to index lookup, count and fetch phases. While the first two phases are relatively constant, the third one clearly grows with the number of nodes.

#### PyEHR with Elasticsearch

**Fig 35:**
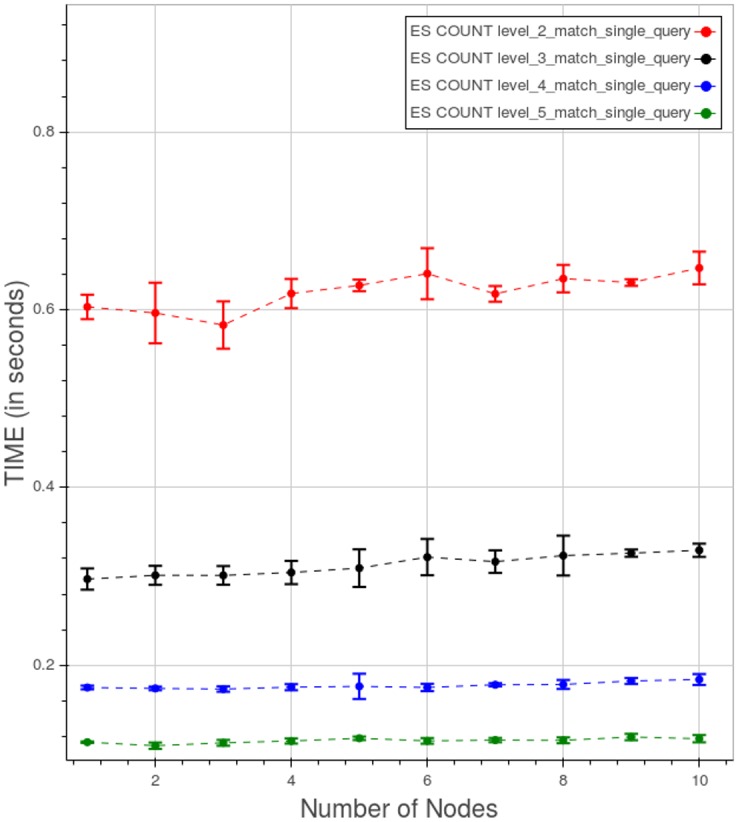
PyEHR with Elasticsearch, CL. Query time for type 1 count query at different levels. Each point is the mean query time, while the bar shows the standard deviation of the measurements.

The results for PyEHR with Elasticsearch for a type 1 count query at different levels are presented in Fig 35. The curves are neatly separated.

**Fig 36:**
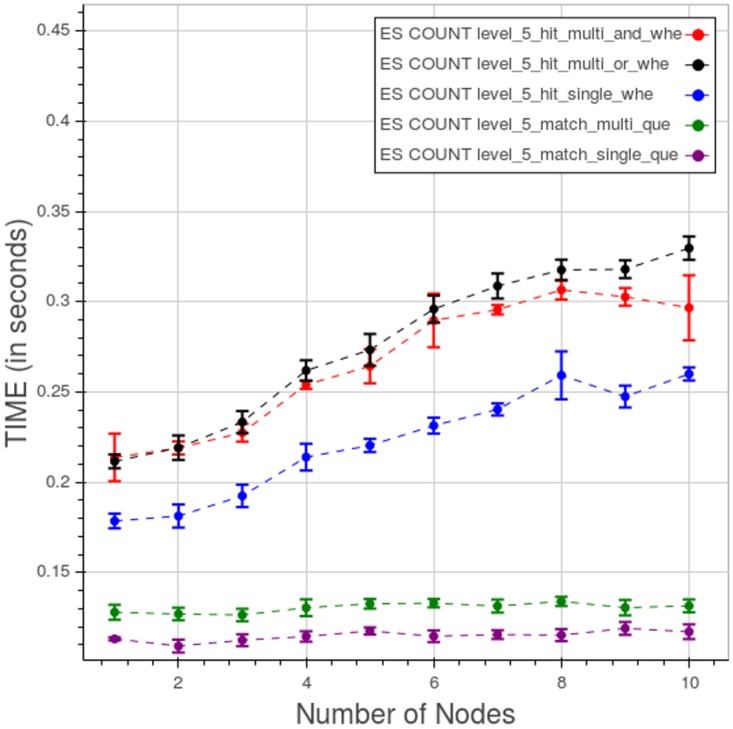
PyEHR with Elasticsearch, CL. Query time for different type of count queries at level 5. Each point is the mean query time, while the bar shows the standard deviation of the measurements.

Fig 36 shows the effect from changing the type of query at level 5. The last three types of query clearly are monotonically increasing with the number of nodes, though the times remain small with respect to both the other database and Apache Hadoop MapReduce.

**Fig 37:**
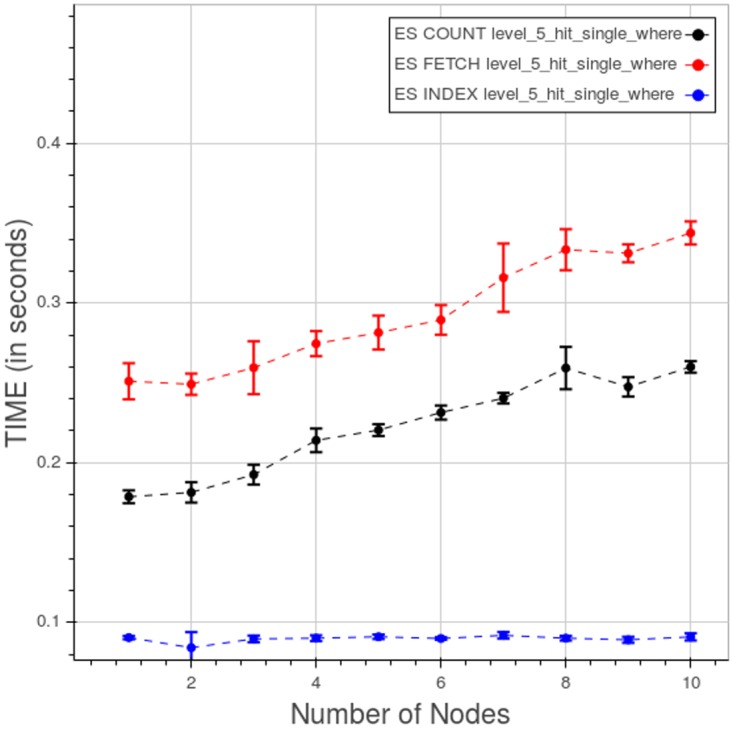
PyEHR with Elasticsearch, CL. Query time spent in the index lookup, count and fetch phases in a type 3 query at level 5. Each point is the mean query time, while the bar shows the standard deviation of the measurements.

Finally, Fig 37 displays the time spent in the index lookup, count and fetch phases for a type 3 query at level 5. While the index lookup time stays relatively constant, both the counting and the fetching times increase as we add nodes and data.

## Discussion

**Fig 38:**
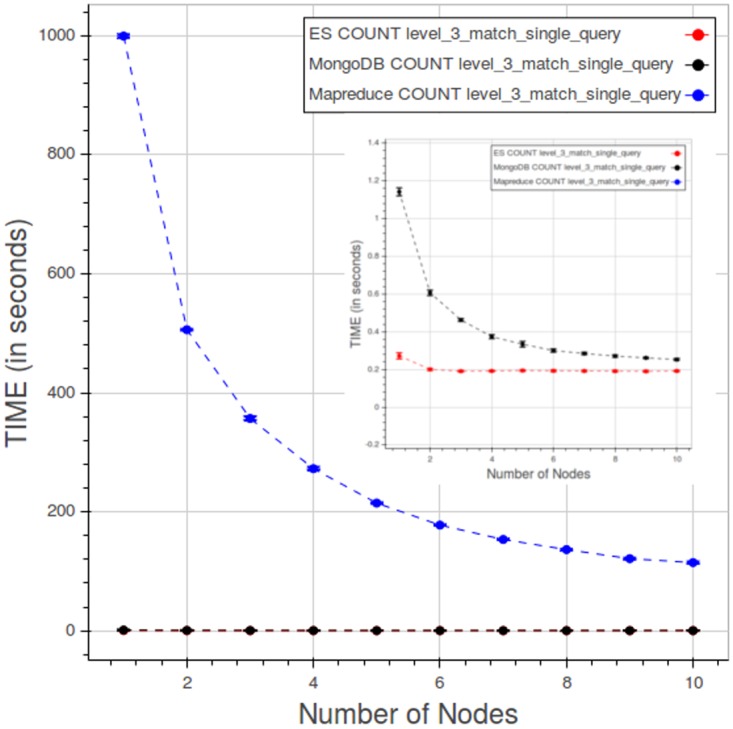
CNR. Results comparing performance for a type 1 query at level 3. Each point is the mean query time, while the bar shows the standard deviation of the measurements.

**Fig 39:**
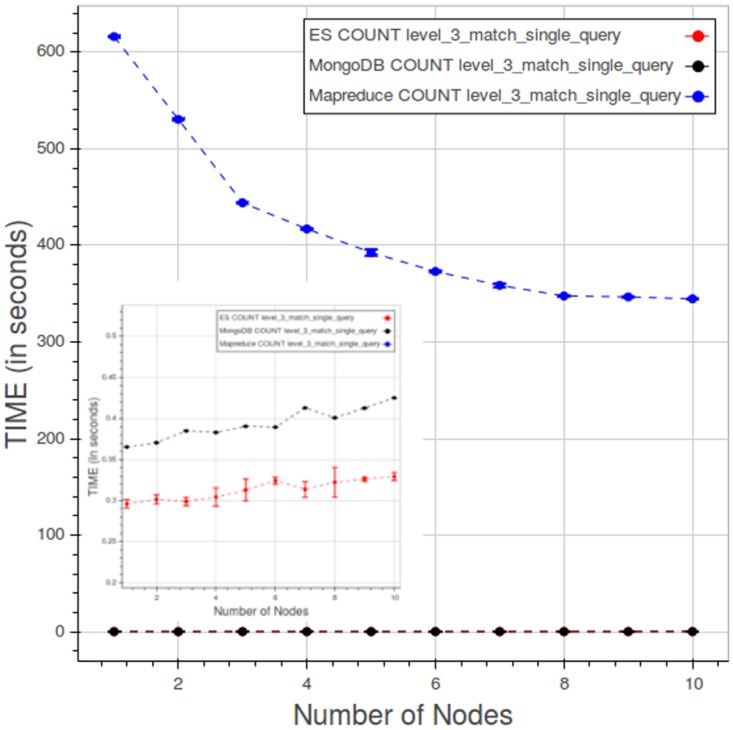
CL. Results comparing performance for a type 1 query at level 3. Each point is the mean query time, while the bar shows the standard deviation of the measurements.

The Figs 38 and 39 compare the PyEHR and Apache Hadoop MapReduce solutions on the CNR and the CL tests, respectively.

Fig 38 refers to a CNR type 1 query at level 4. The expected outcome was that query time should improve as more computing nodes were added, at least to a certain point. This expectation is confirmed by all three curves. Moreover, the results presented in Figs 25 and 29 show that the cost of performing index lookups is constant with respect to the number of nodes, depending solely on the number of data/structures; on the other hand, the counting and fetching times improve as computing nodes are added to the system, though it does not scale indefinitely. The total query times for PyEHR, with any back-end database, and the baseline Apache Hadoop MapReduce are of course very separated –they differ by two to three orders of magnitude.

Fig 39 relates to CL test—specifically, to a type 1 query at level 3. The desired behavior is a constant query time curve where the time value remains the same while simultaneously adding both data and nodes, endlessly. Apache Hadoop MapReduce does better than expected, improving as we add nodes until about eight nodes; then the curve flattens. On the other hand, the performance of PyEHR with either database becomes slightly worse as data and nodes are added; this effect is particularly noticeable in the fetch operation, a behavior that can be inferred by examining Figs 34 and 37. In any case, the overall query times are two to three orders of magnitude smaller than those of Apache Hadoop MapReduce.

We reckon that the structure indexing performed in PyEHR is the primary reason for the huge performance advantage with respect to Apache Hadoop MapReduce in both tests. In fact, the query engine retrieves a list of the structures that match the given query directly from the Index Service, each with their path or paths. This information allows the driver to immediately exclude large portions of the dataset and therefore accelerate considerably the overall query process. In addition Apache Hadoop MapReduce has to run through all parts of the structures whereas PyEHR evaluates only the path or paths indicated by the Index Service for each structure of the list, thus saving additional time. It is important to note that our structures indexing has a low impact on insertion and neglectable storage footprint. PyEHR behaves well with both back-end databases—MongoDB and Elasticsearch. We see a little room for improvement in the parallelization of the index service, though its contribution to the query times in our tests is mostly very small. Admittedly, however, though we eschewed ourselves from optimizing the MongoDB and Elasticsearch bulk insertion configuration parameters, in our tests the time to insert the records is largely in favour of Apache Hadoop MapReduce.

## Conclusion

In this study we have presented PyEHR, a data access layer, designed to help the creation of clinical and biomedical data management systems for secondary use. We have also demonstrated its scalability with both Elasticsearch and Mongodb. In particular, in both its configurations PyEHR outperforms a program written for Apache Hadoop MapReduce on all the query tests conducted. The reasons for its superior performance are to be found in an efficient system of structure indexing and the leverage of high-performance NoSQL database management systems. Future work will be probably focused on upgrading our data access layer to the latest release of Elasticsearch and extending AQL support. Another likely improvement is the parallelization of the index service.

PyEHR is freely available and distributed open source on GitHub at https://github.com/crs4/pyEHR.

### Limitations of this work

We reckon that this work has the following limitations:

- This study does not simulate a real production scenario with concurrent accesses to the system and intermixed insertion, update and query operations. Essentially in our tests the queries are executed sequentially;
- The test datasets are synthetic—not comprised of real EHRs—even though we ensured that they are challenging to query efficiently, maybe more so than real data;
- The queries do not include ORDER BY and TIMEWINDOW clauses as those features are not yet implemented;
- The ACID properties of the system are about the same of the underlying database. We added the external—to the database—versioning support for our EHRs with optional rollback of previous versions;
- The results both for the data insertion and query tests are the product of little of no configurations tweaking so they are not supposed to be the best possible results. There is definitely room for improvement;
- To assess the scalability we used the following software: Apache Hadoop 2.6.0, MongoDB 3.04, Elasticsearch 1.5.0, BaseX 8.0.1;
- With respect to the openEHR implementation, in order to get a fully compliant system there should be a data and query validation service, that can fetch archetypes/template from a local/remote repository, a terminology translator and, optionally, a data templates provider.
